# Development of ELISA against milk haptoglobin for diagnosis of subclinical mastitis in goats

**DOI:** 10.1016/j.heliyon.2021.e06314

**Published:** 2021-02-22

**Authors:** Sarasati Windria, Siti Isrina Oktavia Salasia, Widi Nugroho, Rini Widayanti, Soedarmanto Indarjulianto

**Affiliations:** a Department of Biomedical Sciences, Division of Microbiology, Veterinary Medicine Study Program, Faculty of Medicine, Universitas Padjadjaran, Bandung, Indonesia; b Department of Clinical Pathology, Faculty of Veterinary Medicine, Universitas Gadjah Mada, Jl. Fauna 2, Karangmalang, Yogyakarta, 55281, Indonesia; c Laboratory of Veterinary Public Health, Faculty of Veterinary Medicine, Universitas Brawijaya, Kalisongo, Dau, Malang, East Java, 65151, Indonesia; d Department of Biochemistry, Faculty of Veterinary Medicine, Universitas Gadjah Mada, Jl. Fauna 2, Karangmalang, Yogyakarta, 55281, Indonesia; e Department of Internal Medicine, Faculty of Veterinary Medicine, Universitas Gadjah Mada, Jl. Fauna 2, Karangmalang, Yogyakarta, 55281, Indonesia

**Keywords:** Haptoglobin, Milk, ELISA, Mastitis, Goat

## Abstract

The study described the development of a haptoglobin-based diagnostic tool for mastitis in Ettawa crossbreed goats. Fifty eight milk samples were collected from a flock of goats in Yogyakarta, Central Java, Indonesia. All samples were tested for mastitis using the California Mastitis Test (CMT), Somatic Cell Count (SCC), and Polymerase Chain Reaction (PCR) to identify *Staphylococcus aureus*, *Streptococcus uberis* and *Streptococcus agalactiae*. The presence of haptoglobin mRNA and proteins in the milk somatic cells was detected using Sanger sequencing and SDS-PAGE, respectively. Milk haptoglobin levels were subsequently estimated using an indirect enzyme-linked immunosorbent assay (ELISA) developed in this study. The Receiver Operating Characteristic (ROC) analysis was performed to compare the sensitivity and specificity of CMT, SCC, and the ELISA using the PCR as the reference standard. Kappa test was used to determine the agreement between the three imperfect tests. Results indicated that somatic cells of goat milk expressed a haptoglobin mRNA with a size of 174 bp and two haptoglobin proteins with molecular weights of 18 kDa and 32 kDa. The PCR test showed that 81% of samples were diagnosed positive for mastitis. At a specificity level of 50%, the ROC indicated that the ELISA was more sensitive compared to SCC or CMT (consecutively, 96%, 94%, and 92%). Kappa values between haptoglobin ELISA and CMT or SCC were high (0.84 and 0.81, respectively). This study indicates that somatic cells of goat milk were capable of synthesizing and secreting haptoglobin. Milk haptoglobin can be a potential target for an early detection of mastitis in goats.

## 1 Introduction

Subclinical mastitis reduces milk production, alters milk composition, reduces the hygienic value of milk and impairs the processing properties of milk, thus causes high economic losses in dairy farms [1, 2, 3, 4]. There have been several methods available to diagnose subclinical mastitis, including bacterial culture, polymerase chain reaction (PCR), California mastitis test (CMT), and somatic cell count (SCC) [5, 6, 7, 8].

The cultural examination has been the standard method for identifying bacterial mastitis [9]. Bacterial culture from the milk sample however, only defines the presence of mastitis pathogens but does not provide a measure of the degree of inflammation associated with the infection. The PCR is very sensitive but expensive compared to other tests [10]. The CMT is widely used in screening tests because of easiness in its application but is less sensitive [11]. The SCC is more sensitive than CMT but needs longer labor time [12]. The SCC can increase in normal dairy goats following apocrine secretion in the absence of the infection in the udder, leading to false-positive results [13]. With all limitations occurring in available tests, the development of rapid and sensitive tools for the diagnosis of subclinical mastitis remains open for investigation.

Haptoglobin (Hp) is one major acute phase protein (APP) in mammals. The APPs are generated mainly in the liver. Their concentration in the circulating blood increases significantly during an acute phase reaction (APR) to inflammation in mammals [14, 15, 16]. The APPs are also known to play pivotal roles in the responses to infection, injury, or tumor growth [17].

Studies on mastitis in dairy cows have shown that haptoglobin is detected not only in blood but also in milk. The studies further elucidate its roles in mammalian body responses to diseases [14,16,18,19]. A significant increase of haptoglobin concentrations in cow milk occurred only hours after experimental induction of mastitis [15,16,19,20]. Parallel examination of blood and milk samples of the same cow showed that the increase in haptoglobin concentration in milk occurred earlier than that in blood [19]. These indicate that milk haptoglobin is a potential target of early diagnosis of mastitis in cows.

While milk haptoglobin in cows has been extensively studied, report on haptoglobin production in goat milk has never been published. We hypothesized that during mastitis, haptoglobin is secreted by somatic cells of goat milk and, and haptoglobin is a potential marker for the development of early diagnostic tool of mastitis in goats. The purposes of our study were to investigate the presence of haptoglobin in the somatic cells of goat milk, the expression of mRNA of haptoglobin in the somatic cells, and to develop an indirect ELISA against haptoglobin for the early detection of mastitis in Peranakan Ettawa (PE) goats.

## 2 Materials and methods

### 2.1 Ethical clearance

In this study, we used balb/c mice to produce polyclonal antibodies against goat haptoglobin. Three groups of balb/c mice from the Unit for Animal Testing Services, University of Gadjah Mada were used in this experiment. Each treatment group contained five mice with an average body weight of 20 g. All procedures performed on animals in this study complied with the ethical clearance issued by the Animal Ethics Committee of Universitas Gadjah Mada with registration number 334/KEC-LPPT/X/2015.

### 2.2 Experimental design

This study used lactating Ettawa-Kacang crossbred goats (Peranakan Ettawa, PE). Kacang goat is an indigenous breed of goat of Indonesia. The study was conducted in a PE goat farm in the Sleman region, Yogyakarta, Central Java, Indonesia. As many as 58 does, showing no clinical mastitis, with the lactation period ranging from two to five months, were selected randomly for the study. A milk sample of each doe was obtained from one half of the udder. The absence of clinical mastitis was deemed when there was no observed inflammation on the udder, no clump in the milk discharged, and the goat did not show pain reflex during milking. Milk samples collection was conducted aseptically. The first few milk discharges were discarded, and 15 mL of milk samples were collected separately in sterile test tubes, stored in a container at 4 °C temperature and transported immediately to the laboratory [21]. The collected samples were divided into aliquots aseptically for further analyses. All laboratory tests were conducted at the Clinical Pathology Laboratory, Faculty of Veterinary Medicine, Universitas Gadjah Mada, Yogyakarta, Indonesia.

### 2.3 California mastitis test (CMT)

The CMT test was conducted following the instruction from the manufacturer (CMT, Jѳrgen Kruuse, Denmark). The test scores are ranging from zero (negative test) to positive three (+3) according to its increasing viscosity. The description of reaction and CMT score were in accordance with the criteria of the International Dairy Federation (IDF) [22].

### 2.4 Somatic cell count (SCC)

The SCC test was conducted following the procedure described elsewhere [23, 24, 25, 26, 27]. A total of 10 μl milk sample was spread on an area of 1 cm^2^ on an object glass. The spread milk sample was aerated to let it dry and was fixed on a Bunsen fire. The layer of milk fat was eliminated using 70% alcohol ether for two minutes. The spread milk sample was stained with methylene blue for 1–2 min, rinsed using 96% alcohol, and was let dry. The spread milk sample was examined under 1000x microscopic magnification in immersion oil, and cells were counted. A cell larger than 10 μm containing nucleus was considered an epithelial cell.

### 2.5 Bacterial identification in milk samples by PCR

Identification of the bacteria by PCR was conducted directly from raw milk without prior bacterial culture, following method described by Ahmadi et al., (2010) [28]. The species of bacteria were identified from milk samples by PCR of the 23S rRNA gene of *Staphylococcus aureus*, GSub gene of *Streptococcus uberis*, and GSyds gene of *Streptococcus dysgalactiae* [[29], [30], [31]]. The primers and cycle programs are shown in Table 1. The reaction mixture (25 μL) contained 1 μL primer 1 (20 pmol) and 1 μL primer 2 (20 pmol; Invitrogen, USA). The DNA of the bacterial isolates was prepared with the QIAamp DNA mini kit (Qiagen, Germany) following the instruction from the manufacturer. The amplification of the genes was carried out with a thermal cycler (Mastercycler, Eppendorf, Germany).

**Table 1:** PCR program used for detecting *S. aureus*, *Str. uberis*, *Str. dysgalactiae* and haptoglobin.

Bacteria/protein detected	*Staphylococcus aureus*	*Streptococcus uberis*	*Streptococcus dysgalactiae*	Haptoglobin
Target DNA/RNA	23SrRNA	GSub	GSdys	mRNA
Primer forward (5-3)	ACGGAGTTACAAAGGACGAC	ACGGAGTTACAAAGGACGAC	AGCTGTGGATTGTCCTTTGG	GTCTCCCAGCATAACCTCATCTC
Primer reverse (5-3)	AGCTCAGCCTTAACGAGTAC	AGCTCAGCCTTAACGAGTAC	TCGCTCGCTCACCTTAGAAT	AACCACCTTCTCCACCTCTACAA
Cycles	37	35	35	37
Pre-denaturation	94 °C, 300 Sec	95 °C, 900 Sec	95 °C, 900 Sec	95^○^C, 60 sec
Denaturation	94 °C, 40 Sec	94 °C, 60 Sec	94 °C, 60 Sec	94^○^C, 40 sec
Annealing	64 °C, 60 Sec	58 °C, 60 Sec	58 °C, 60 Sec	52^○^C, 20 sec
Elongation	72 °C, 75 Sec	72 °C, 60 Sec	72 °C, 60 Sec	72^○^C, 20 sec
Final extension	72 °C, 300 Sec	72 °C, 600 Sec	72 °C, 600 Sec	72^○^C, 300 sec
Reference	(Salasia et al., 2011) [28]	(Raemy et al., 2013) [29]	(Raemy et al., 2013) [29]	(Altschul et al., 1990) [33]

The PCR products were separated by gel electrophoresis in a 1.5% (w/v) agarose gel (Roth, Germany) in 0.5×TBE buffer (containing a mixture of tris base, boric acid, and EDTA. A 100 bp DNA ladder (Geneaid, Taiwan) was used as a size marker. The resulting bands were visualized using FloroSafe (1st Base, Singapore) staining under UV trans-illumination.

### 2.6 Identification of haptoglobin in milk somatic cells

Milk somatic cells were isolated from 200 mL freshly collected goat milks which were clinically normal but shown positive on CMT test. Briefly, milk samples were centrifuged at 1,000 g for 20 min at 4 °C. After removing the fat layer and supernatant, the pellet was washed twice with phosphate buffer saline (PBS) to remove protein from the somatic cells and maintained on the ice. The cell pellet was then aliquoted into two tubes for haptoglobin protein profiling and mRNA analysis and stored at -80 °C until analysis [32].

Extraction of total protein of cells was conducted using a lysis buffer containing 0.3% Triton X-100 in PBS, a sonication at 4 °C for 5 min, and centrifugation at 10,000 g for 10 min at 4 °C. The remaining supernatant was stored at –20 °C until used in an SDS-PAGE analysis [33]. Haptoglobin was purified from the somatic cells by SDS-PAGE followed by electroelution as previously described [33]. Supernatant of the somatic cells was subject to SDS-PAGE electrophoresis on vertical slab gel. The polyacrylamide gel consisted of 12% resolving gel and 5% stacking gel. The electrode buffer was 0.3% (w/v) Tris, 1.44% (w/v) glycine and 0.1% (w/v) SDS (pH 8.3).

The sample was preheated at 100 °C for 2 min in a buffer containing 12mM Tris–HCl, 0.4% SDS, 5% glycerol, 2.9 mM 2-mercaptoethanol, and 0.02% bromphenol blue, pH 6.8, before loading to the gel. Each well was loaded with 12 μl sample buffer and the somatic cell supernatant with a ratio of 1:4. The samples were run in the gel for 1.5 h at 100 V and the gel was subsequently stained using Coomassie Brilliant Blue. The marker for the molecular weight of proteins was a molecular-mass standard containing 13 pre-stained proteins (3.5–245 kDa) provided by 1^st^ BASE (Singapore).

The presence of haptoglobin was determined using the relative mobility (Rf) values, which corresponded with the molecular weight of haptoglobin. Bands presumptive of haptoglobin from somatic cells of goat milk were detected at molecular weights of 32 kDa (β-haptoglobin) and 18 kDa (α-haptoglobin). The proteins with these molecular weights were cut and were put into a dialysis pouch to which 2 mL TBE (Tris/Borate/EDTA) buffer was added. Electrophoresis apparatus was connected to a power supply of 100V, 300mA, and protein was eluted from the gel (the solution turned blue and the gel turned transparent). The purified protein was aspirated using a 1 mL syringe and stored at -20 °C.

The concentration of purified haptoglobin was measured by Bio-Rad protein assay. As much as 2 μL of protein elution was added into 798 μL of distilled water and 200 μL of Bio-Rad Protein Assay solution (Bio-Rad, USA). Spectrophotometer UV-1700 (Shimadzu, Japan) was used to read the absorbance of the solution at a wavelength of 595 nm.

### 2.7 Identification of mRNA encoding haptoglobin in milk somatic cells

A high purity RNA isolation kit (Roche, Germany) was used to extract total RNA from somatic cells of the goat milk according to the manufacturer's protocol. Extracted RNA was stored in the freezer at 80 °C. Haptoglobin gene was amplified using one-step RT-PCR Kits (Roche, Germany). Haptoglobin gene primer pair was used to produce 174 bp amplicon. Primers used for amplification of genes encoding haptoglobin consist of the forward primer 5′ GTCTCCCAGCATAACCTCATCTC 3′ and reverse 5′ AACCACCTTCTCCACCTCTACAA 3′. The thermocycling program used is described in Table 1. It consisted of 37 cycles of pre-denaturation (95 °C for 1 min), denaturation (94 °C for 40 s), annealing (55 °C for 20 s), elongation (72 °C for 20 s), post-elongation (72 °C for 5 min) [4]. Real time (RT)-PCR product was then run for electrophoresis on 1 % agarose gel, visualized using Fluoro safe (1st BASE, Singapore) staining under UV trans-illumination and compared with marker 100 bp DNA Ladder (Bioline, USA). The PCR products of RT-PCR were sequenced by 1^st^ BASE for DNA sequencing with Sanger Sequencing Method (1^st^ BASE, Singapore). A nucleotide sequence homology analysis was performed through the National Center for Biotechnology Information (NCBI) BLAST Network Service according to the algorithm of Altschul et al. (1990) [34].

### 2.8 Development of the indirect ELISA to detect haptoglobin in goat milk

Polyclonal antibodies against haptoglobin was produced using balb/c mice. Three groups of balb/c mice were used in this experiment. Each group contained five mice with an average weight of 20 g. Each mouse in group one was injected intraperitoneally with 10 μg of purified β–haptoglobin emulsified in 875μL of Freund's complete adjuvant (Sigma Aldrich, USA). Each mouse in group two was injected intraperitoneally with 10 μg of purified α–haptoglobin in 875μL of Freund's complete adjuvant (Sigma Aldrich, USA). Mice in the group three were injected with phosphate buffer saline intraperitoneally as the control group. Boosting injections were given to each of mice in treatment groups repeatedly at an interval of seven days by injecting a 10 μg of purified β or α haptoglobin in Freund's incomplete adjuvant (Sigma Aldrich, USA) intraperitoneally. Mice in the control group were given PBS as a booster. To test the development of antibody against haptoglobin, all mice were bled from retroorbital plexus three days after each booster immunisation. Blood was allowed to clot at room temperature, and after the centrifugation at 2000 g, aliquots of the antiserum was stored at -20 °C. Absorbance value and concentration of polyclonal antibody, as well as binding specificity of the polyclonal antibody against haptoglobin, were measured using indirect ELISA method [35].

### 2.9 The development of procedure of indirect ELISA to detect goat milk haptoglobin

Indirect ELISA-specific binding showed that the average absorbances of two independent experiments were 1.081 in the β-haptoglobin group, 0.447 in the α-haptoglobin group and 0.422 in the control group. These corresponded to the concentrations of a purified polyclonal antibody of 9.92 mg/mL in the β-haptoglobin group, 6.24 mg/mL in the α-haptoglobin group and 6.71 mg/mL in the control group. Due to this higher binding level, the purified polyclonal antibody against β-haptoglobin was used to detect and measure the concentration of haptoglobin in goat milk samples.

Goat milk sample was treated to remove fat and somatic cell contents and was used to coat Microtiter plate (Nunc-immunoplate, Maxisorp, Roskilde, Denmark) at a dilution of 1/40,000 in 100 μL of 0.2 M NaHCO_3_ and 0.2 M Na_2_HCO_3_, and was incubated at 37 °C for 1 h. The plate was blocked with 1% of bovine serum albumin (BSA) in 200 μL of phosphates buffer saline at pH 7.0 at 37^ο^C for 1.5 h. To each well, 100 μL of the polyclonal antibody (1/100 dilution) was added and incubated at 37 °C for 1 h. After three washes, 100 μL of the second antibody (IgG anti-mouse alkaline phosphatase, Sigma, USA) in 1/16,000 dilution was added into wells of the plate and was incubated at 37 °C for 1 h. After washed three times, the wells were filled with 150 μL of 4-nitrophenyl-phosphate substrate (1 mg/mL in substrate buffer solution) and incubated at 37 °C for 15 min. The absorbance was subsequently read in an ELISA reader at 405 nm (Zenix-320). The reading for each of the samples were performed in triplicate. The absorbance was converted into concentration using a linear regression formula developed previously using the absorbance of purified haptoglobin [19,32,33,36,37].

### 2.10 Statistical analyses

The coefficient of variation (CV) of the tests for each of 58 samples were calculated by dividing the standard deviation of the triplicate tests by the mean. A ROC curve was developed for the three diagnostic tests (Haptoglobin ELISA, SCC and CMT) using the PCR result as the reference standard, and the Area Under Curves (AUC) of the tests were compared. A 50% specificity was set to compare sensitivity of the tests against the reference standard. Kappa statistic, to determine diagnostic aggreement among the three tests, was calculated at a 95% confidence limit. A correlation analysis among the three tests was calculated at a 95% confidence limit. Data of SCC for correlation analysis was presented in 10-points simple moving average (SMA). The ROC and kappa statistic calculations were performed in IBM-SPSS 25 (NY, USA).

## 3 Results

The PCR detected DNA of the three bacterial species in 87.9% (51/58) of the milk samples. *Staphylococcus aureus* were detected in 82.8% (48/58) samples; 62.5% (30/48) of them were catalase negative *Staphylococcus aureus*. *Streptococcus uberis* was detected in one sample (1.7%) and *Streptococcus dysgalactiae* was detected in two samples (3.4%). There was no co-infection with the three bacteria detected in any of samples.

The CMT test showed that eight samples were tested negative (13.6%), 14 samples were tested +1 (23.7%), 14 samples were tested +2 (23.7%), and 23 samples were tested +3 (39.0%). Somatic cell count showed that the range of the number of somatic cells in all milk samples was 0.35–11.86 × 10^6^ cells/mL.

**Figure 1:**
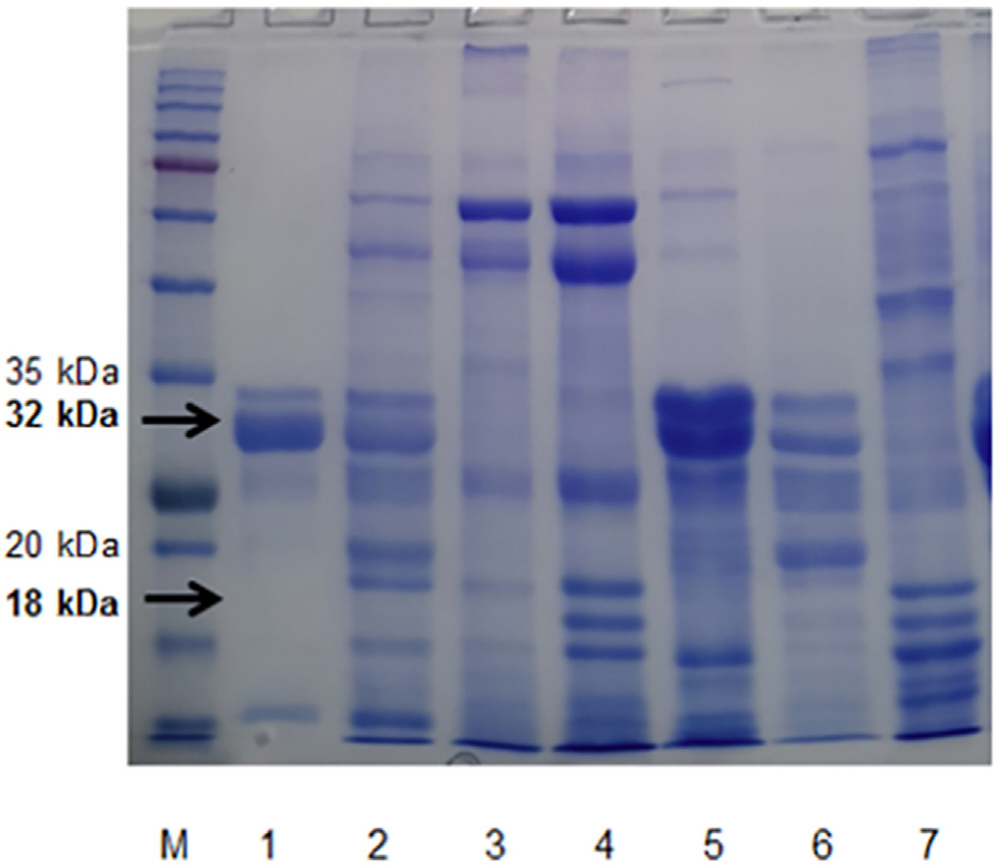
Haptoglobin profile of mastitis milk goats. Lane 1: Kl02, lane 2: Kl06, lane 3: Kl01, lane 4: Kl05, lane 5: 10Bs, lane 6: Sk01, lane 7: Sk02. The SDS-PAGE analysis detected two bands of haptoglobin molecules with molecular weights of 32 kDa and 18 kDa. M: Marker protein (1^st^ BASE, Singapore), whole protein concentration 1,331, SDS-PAGE 12%.

The lowest concentration of whole protein of somatic cell in the samples was 1.35 μg/μL (range: 1.35 μg/μL – 14.2 μg/μL). This concentration was the basis for the lower limit running of SDS-PAGE for haptoglobin profiling. The SDS-PAGE analysis detected two bands of haptoglobin molecules with molecular weights of 32 kDa and 18 kDa (Figure 1, Supplementary Figure 1). The highest concentration of the 32 kDa haptoglobin among samples was 0.74 μg/μL. The highest concentration of the 18 kDa haptoglobin was 0.76 μg/μL. The range of total haptoglobin concentration in milk was 68.45–949.42 mg/mL.

**Figure 2:**
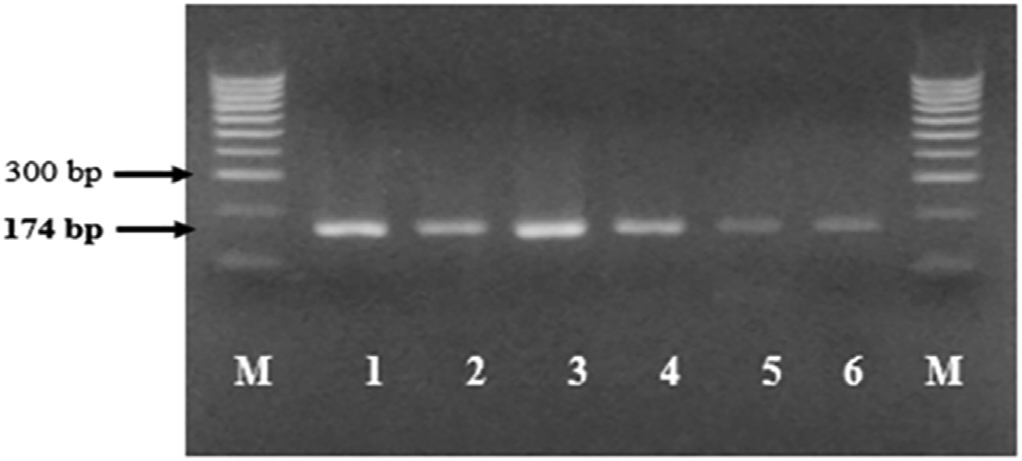
Qualitative RT-PCR mRNA Hp (174 bp) from somatic cells of goat milk. Lane 1: Kl01, lane 2: Kl04, lane 3: Kl05, lane 4: Kl06, lane 5: Sk01, lane 6: Sk02, M: Marker DNA (Bioline 100 bp).

**Figure 3:**
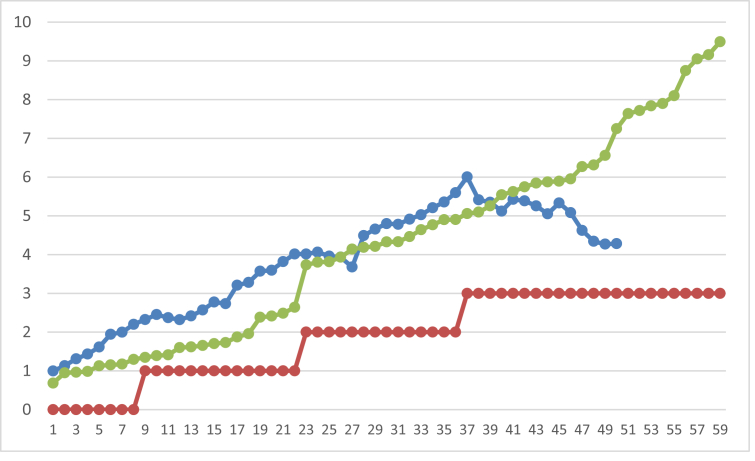
Correlation between haptoglobin concentration, Somatic Cell Counts and the scores of California Mastitis Test of goatmilk.  SCC (x10^6^, 10 SMA),  CMT and  Haptoglobin (x10^−2^ mg/mL).

The PCR detected mRNA encoding haptoglobin in somatic cells of goat milk that were tested positive for mastitis by CMT test. The size of the haptoglobin gene corresponded to the expected size of 174 bp (Figure 2, Supplementary Figure 2). The DNA sequences of samples showed 99% homology with the Gene Bank database of *Caprae hircus* haptoglobin gene sequences with the accession number of XM_005692202.1 and FJ194972.1 (Table 2). Based on the cDNA sequence, the caprae haptoglobin β-chain comprises 245 amino acid residues. Correlation analysis showed that haptoglobin has a high positive correlation with CMT (R: 0.92) and SCC (R: 0.89). However, at a level of CMT +3, the SCC tended to decline while the haptoglobin concentration steadily increased (Figure 3).

**Table 2:** The sequence of cDNA RT-PCR from mastitis goat milk somatic cells.

Code	Sequence	Result
Kl04	TTGTCTCCCAGCATAACCTCATCTCGGGAGCCACACTCATCAATGAACGATGGCTCCTCACCACAGCTAAAAATCTCTACCTGGGTCACACTAGTGACAAAAAAGCAAAGGACATCACTCCTACTTTAAGACTCTATGTGGGGAAGAACCAGCTTGTAGAGGTGGAGAAGGTGGTTA	99% Identity
		Predicted: Capra hircus haptoglobin (HP), mRNA
		Capra ibex haptoglobin mRNA, complete cds
Kl06	TTGTCTCCCAGCATAACCTCATCTCGGGAGCCACACTCATCAATGAACGATGGCTCCTCACCACAGCTAAAAATCTCTACCTGGGTCACACTAGTGACAAAAAAGCAAAGGACATCACTCCTACTTTAAGACTCTATGTGGGGAAGAACCAGCTTGTAGAGGTGGAGAAGGTGGTTA	99% Identity
		Predicted: Capra hircus haptoglobin (HP), mRNA
		Capra ibex haptoglobin mRNA, complete cds
Sk01	TTTGTCTCCCAGCATAACCTCATCTCGGGAGCCACACTCATCAATGAACGATGGCTCCTCACCACAGCTAAAAATCTCTACCTGGGTCACACTAGTGACAAAAAAGCAAAGGACATCACTCCTACTTTAAGACTCTATGTGGGGAAGAACCAGCTTGTAGAGGTGGA	99% Identity
		Predicted: Capra hircus haptoglobin (HP), mRNA
		Capra ibex haptoglobin mRNA, complete cds
SK02	TGTCTCCCAGCATAACCTCATCTCGGGAGCCACACTCATCAATGAACGATGGCTCCTCACCACAGCTAAAAATCTCTACCTGGGTCACACTAGTGACAAAAAAGCAAAGGACATCACTCCTACTTAAGACTCTATTGAGTAGCTAC	99% Identity
		Predicted: Capra hircus haptoglobin (HP), mRNA
		Capra ibex haptoglobin mRNA, complete cds
10Bs	TGTCTCCCAGCATAACCTCATCTCGGGAGCCACACTCATCAATGAACGATGGCTCCTCACCACAGCTAAAAATCTCTACCTGGGTCACACTAGTGACAAAAAAGCAAAGGACATCACTCCTACTTAAGACTCTATTGAGTACGTTAC	99% Identity
		Predicted: Capra hircus haptoglobin (HP), mRNA
		Capra ibex haptoglobin mRNA, complete cds

**Figure 4:**
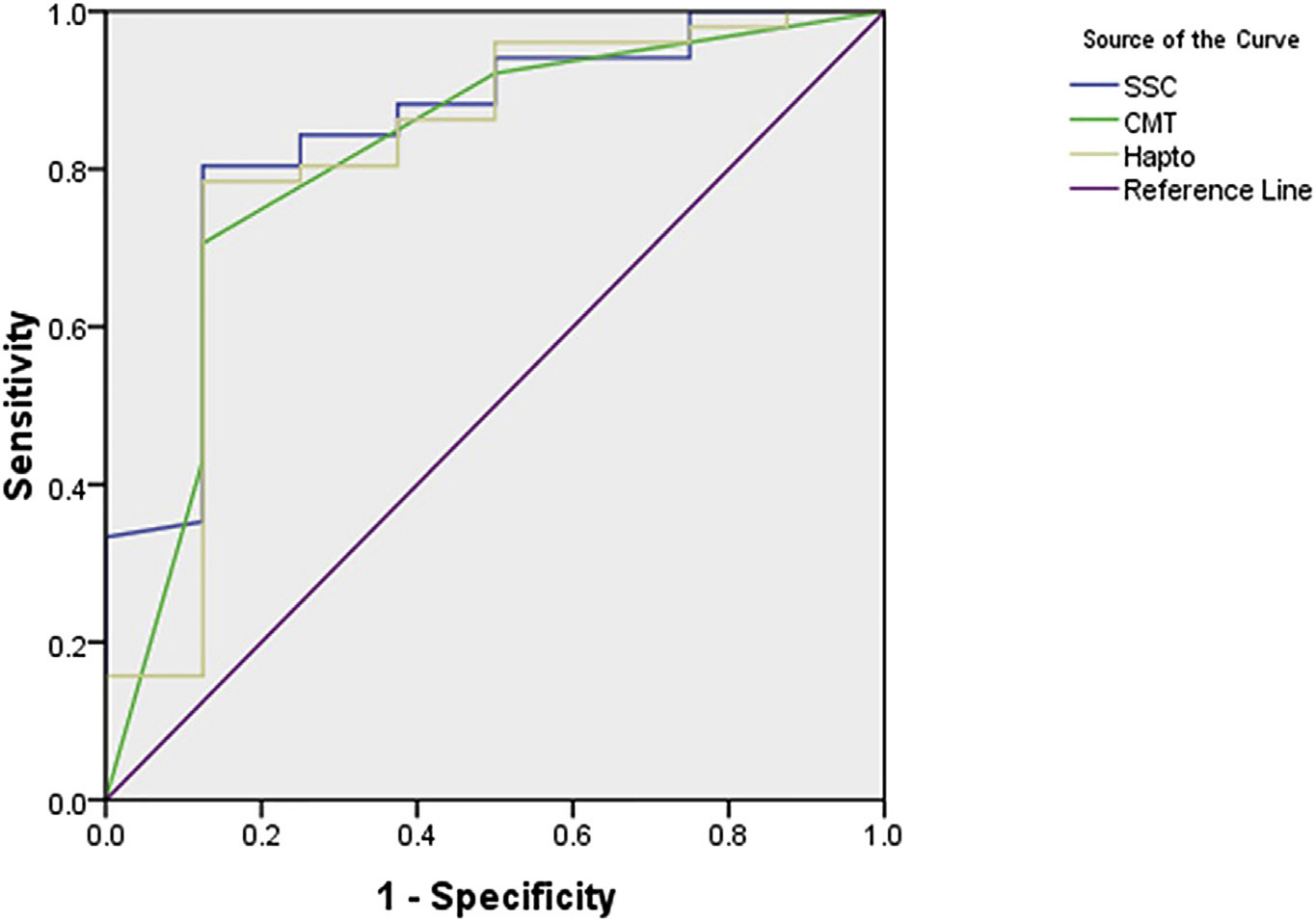
The area under the curve of ROC analysis of three diagnostic methods to detect masititis in goats. SCC (Somatic Cell Counts), CMT (California Mastitis Test), Hapto (ELISA against haptoglobin).

The CV for tests with the absorbances of lower than 1.4 (corresponded to a haptoglobin concentration of 155.61 mg/mL) were consistently below 10% (range: 0.2–9.9%), while the CV of tests with the absorbance of 1.4 or higher showed more varied SC value (range: 1.1–17.8%). The ROC analysis showed that the AUC of haptoglobin test was in between that in SCC and CMT (0.814, 0.844, and 0.812 consecutively, *p* < 0.01). Under the setting of 50% specificity, indirect ELISA test against haptoglobin appeared slightly more sensitive than SCC or CMT tests (Figure 4). The cut-off of haptoglobin concentration for a specificity level of 50% of the ELISA was 116.34 mg/mL. The cut-off value of CMT for the 50% specificity level was +1, while cut-off value for SCC at 50% specificity was 0.97 × 10^6^ cells/mL. Kappa test using these cut-off values showed that the haptoglobin ELISA had a high agreement with both SCC and CMT tests (Kappa: 0.81 and 0.84, respectively).

## 4 Discussion

This study reported for the first time, expression of mRNA encoding haptoglobin in somatic cells of goat milk. This study confirmed that haptoglobin protein and the mRNA were detected in somatic cells of goat milk and indicated that these cells secrete the protein into goat milk. This finding supports studies that reported that in ruminant species, haptoglobin is synthesized by somatic cells in the mammary gland, and its presence in milk is not due to leakage through the blood milk barrier alone [24,33,38].

This study indicated that haptoglobin was produced and secreted by somatic cells of goat milk during the early phase of udder infections with bacteria such as *Staphylococcus aureus, Streptococcus uberis*, and *Streptococcus dysgalactiae*. This finding further supported previous studies that reported that haptoglobin was detectable in serum and milk of ruminants suffering from mastitis [12,[39], [40], [41], [42]]. In this study, Staphylococci play role in causing infection and increasing the concentration of haptoglobin as an indicator of inflammation. *Staphylococcus aureus* were detected in 87.9% and coagulase negative staphylococci (CNS) were detected in 62.5% of samples. These results indicate that the CNS has potential to invade the udder and induce an inflammatory reaction. The CNS have several virulence factors including cytolysins which have synergistic roles such as β-hemolysin, leukotoxins, metalloprotease cytotoxins, Staphylococcal enterotoxins and Staphylococcal enterotoxins-like superantigens [43]. The results of other studies support this study that high concentrations of haptoglobin in serum or milk occur when animals suffer from mastitis [39,40,44].

Molecular weights of haptoglobin detected in goat milk were similar to those detected in sera, with two polypeptide forms with molecular weights of 32 kDa (β-chain) and 18 kDa (α-chain) [33,45,46]. The β-chain haptoglobin could be a potential biomarker for mastitis diagnosis.

The result of haptoglobin ELISA in this study was in high aggreement with SCC and CMT. However the SCC declined after a certain point at high haptoglobin concentration or high CMT score, while the values of latter two tests showed steady increases. Other study indicated similarly that SSC of goat milk declined after three weeks postinfection [47]. The reasons might be that while SCC counts only intact cells, CMT detects DNA material of intact cells, ruptured somatic cells as well as bacterial cells in the milk. The difference between SCC and APP (haptoglobin) was in the udder release mechanism [33,48]. Leukocytes migrate actively through adhesion molecules. Haptoglobin releases in the udder due to passive leakage from the blood into the milk, increasing permeability at the inflammatory site and producing locally in the udder environment [18,49]. These may explain why the CMT scores remained high while SCC declined after a certain point. Similarly, the synthesis and secretion of haptoglobin into milk continued by remaining alveolar cells, by leukocytes in the inflamed area, and from leakage of blood vessels [24,33,38,50]. These resulting in a higher concentration of goat milk haptoglobin in animals with severe mastitis.

The ROC curve showed that the haptoglobin test has an area under the curve similar to that in CMT and SCC tests. The ROC curve indicated that the haptoglobin test could have had a higher sensitivity under the specified specificity setting. Further, the Kappa test among haptoglobin, CMT, and SCC assay showed high agreement, so as the correlation value. Similarities in the type of detection target in the three methods may have led to the high agreement and correlation values, especially during the mild to moderate level of SCC [37].

The CV of the haptoglobin ELISA was less than ten percent in lower concentration haptoglobin. It indicates that the ELISA could yield consistent results when used to diagnose mastitis under the cut-off value set up in this study. However, the use of this ELISA to determine a higher concentration of haptoglobin may not be that accurate, indicated by the higher CV value in the higher absorbance level of the test. However, this condition may not compromise the use of haptoglobin as the diagnostic marker because the aim of the study was to detect a lower concentration of haptoglobin as the indicator of acute mastitis.

## 5 Conclusions

In conclusion, the presence of the mRNA and protein of haptoglobin in the milk somatic cells of Ettawa crossbred goat indicates that these cells synthesize haptoglobin. Haptoglobin could be a suitable marker for the development of diagnostic test of subclinical mastitis in goats.

## Declarations

### Author contribution statement

Sarasati Windria: Performed the experiments; Contributed reagents, materials, analysis tools or data; Wrote the paper.

Siti Isrina Oktavia Salasia: Conceived and designed the experiments, Analyzed and interpreted the data; Contributed reagents, materials, analysis tools or data; Wrote the paper.

Widi Nugroho: Analyzed and interpreted the data; Wrote the paper.

Rini Widayanti, Soedarmanto Indarjulianto: Performed the experiments.

### Funding statement

This work was supported by 10.13039/501100010447Ministry of Research, Technology and Higher Education of the Republic of Indonesia (No. 279/LPPM/2015) and (No. 1703/UN1/DITLIT/DIT-LIT/LT/2018).

### Data availability statement

Data will be made available on request.

### Declaration of interests statement

The authors declare no conflict of interest.

### Additional information

No additional information is available for this paper.
