# Supplementary material for: Development of ELISA against milk haptoglobin for diagnosis of subclinical mastitis in goats
Source: Heliyon. 2021 Feb 22;7(2):e06314. doi: 10.1016/j.heliyon.2021.e06314 (PMC7907475; doi:10.1016/j.heliyon.2021.e06314)
Supplement: Supplementary Figures [file mmc1.docx]

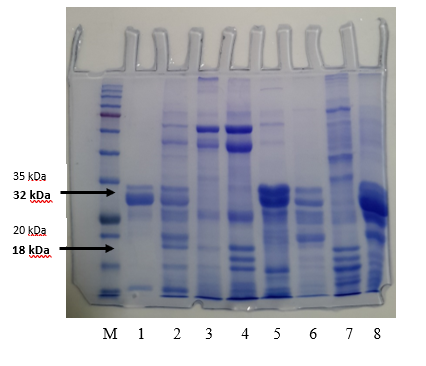


Supplementary Figure 1. Haptoglobin profile of mastitis milk goats. Lane 1: Kl02, lane 2: Kl06, lane 3: Kl01, lane 4: Kl05, lane 5: 10Bs, lane 6: Sk01, lane 7: Sk02, lane 8: Kl04. The SDS-PAGE analysis detected two bands of haptoglobin molecules with molecular weights of 32 kDa and 18 kDa. M: Marker protein (1^st^ BASE, Singapore), whole protein concentration 1,331, SDS-PAGE 12%.


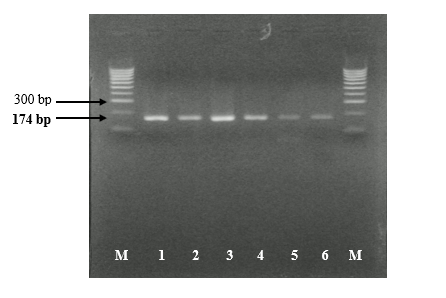


Supplementary Figure 2. Qualitative RT-PCR mRNA Hp (174 bp) from somatic cells of goat milk. Lane 1: Kl01, lane 2: Kl04, lane 3: Kl05, lane 4: Kl06, lane 5: Sk01, lane 6: Sk02, M: Marker DNA (Bioline 100 bp).
